# Unlocking the three-node regulatory axis: polyamines, antioxidants, and photosynthetic protection in stressed fruit crops

**DOI:** 10.3389/fpls.2026.1984273

**Published:** 2026-10-21

**Authors:** Masood Jan, Carlos Eduardo Aucique-Perez, Matthew Mattia, Zafar Iqbal, Muhammad Adnan Shahid

**Affiliations:** 1 Horticultural Sciences Department, North Florida Research and Education Center, Institute of Food and Agricultural Sciences, University of Florida, Quincy, FL, United States; 2 United States Horticultural Research Laboratory, United States Department of Agriculture, Agricultural Research Service, Fort Pierce, FL, United States; 3 Central Laboratories, King Faisal University, Al-Ahsa, Saudi Arabia

**Keywords:** polyamines, abiotic stress, antioxidant defense, *Arabidopsis*, Calvin cycle, fruit crops, photosynthesis

## Abstract

Abiotic stresses, including drought, salinity, heat, and cold, disrupt cellular redox homeostasis and impair photosynthesis in fruit crops, directly threatening vegetative growth, fruit set, and marketable yield. While polyamines (PAs), mainly putrescine, spermidine, and spermine, are well-known mediators of environmental stress tolerance, their precise molecular roles in fruit crops remain fragmented. This review conceptualizes PA-mediated stress tolerance through a novel “three-node regulatory axis”. The first node governs cellular PA homeostasis through a tightly coordinated network of biosynthesis (via arginine decarboxylase [ADC], S-adenosylmethionine decarboxylase [SAMDC], and spermidine/spermine synthases [SPDS/SPMS]) and catabolism (via diamine and polyamine oxidases [DAO/PAO]). The second node involves antioxidant activation, where controlled PA catabolism generates signaling levels of hydrogen peroxide (H_2_O_2_) that prime enzymatic and non-enzymatic defense mechanisms and preserve cellular and chloroplast redox poise. Beyond direct antioxidant activation, PA-dependent redox signals can interface with Ca²^+^-dependent pathways, MAPK cascades, nitric oxide, and hormone signaling to regulate stress-responsive gene expression. The third node encompasses photosynthetic protection, maintaining thylakoid membrane stability, protecting photosystem II (PSII) from photoinhibition, regulating stomatal behavior, and supporting carbon assimilation under stress. Although the model plant *Arabidopsis thaliana* provides a valuable reference framework, direct molecular extrapolation to fruit crops is limited. Fruit crops retain the ornithine decarboxylase (ODC) pathway (which is absent in *Brassicaceae*), undergo complex perennial woody growth, exhibit fruit-specific hormone crosstalk (particularly PA–ethylene interactions during ripening), and face prolonged field stress conditions. Currently, critical molecular gaps remain in fruit crop research, particularly regarding the direct regulation of Calvin cycle gene expression (such as Rubisco, GAPDH, and SBPase), the functional characterization and regulation of PA transport systems across fruit crops, the direct validation of chloroplast-localized PA biosynthesis, and the upstream transcription factor modules coordinating these responses. To transition the field from physiological association to causal molecular mechanism, future research must prioritize time-resolved, multi-endpoint experimental designs integrating PA profiling, gas exchange, and transcriptomic or proteomic analyses in identical plant systems combined with long-term field evaluation to translate these physiological insights into robust, climate-resilient fruit production.

## 1 Introduction

Abiotic stress is now one of the primary biological limitations to achieving stable fruit production (Cosmulescu, 2026; Manzoor et al., 2023). Drought, salinity, heat, cold, high light, and oxidative stress can severely impair vegetative growth, disrupt pollination and flowering, affect fruit set, and ultimately reduce fruit size, yield, and overall quality (Cosmulescu, 2026). These adverse effects are particularly critical in fruit crops because fruit development is highly dependent on a continuous and robust supply of assimilates from photosynthetically active source tissues. Under the ongoing challenges of climate change, these stress events are becoming increasingly frequent, severe, and complex. In field environments, fruit trees are frequently exposed to combined stresses such as drought occurring with heat, or salinity co-occurring with high light and oxidative stress. Consequently, developing and improving stress resilience in fruit crops requires a precise, mechanistic understanding of how photosynthetic systems are protected, rather than simply measuring final yield losses (Lobell and Gourdji, 2012; Manzoor et al., 2023; Nidhi et al., 2025).

At the cellular level, different environmental stresses often converge on a common pathological bottleneck: the disruption of redox homeostasis (Foyer and Noctor, 2005; Mittler, 2006). Stress conditions limit CO_2_ assimilation, disturb electron transport, and cause overproduction of reactive oxygen species (ROS), such as superoxide radicals (O_2_•−), H_2_O_2_, and hydroxyl radicals (Mittler, 2006; Gill and Tuteja, 2010). When the ROS production exceeds the capacity of the antioxidant system, oxidative damage occurs in membranes, proteins, pigments, and nucleic acids (Gill and Tuteja, 2010; Mittler et al., 2011). The photosynthetic apparatus is especially vulnerable because thylakoid membranes contain oxidizable lipids, PSII requires continuous repair of the D1 reaction center protein, and Calvin cycle enzymes such as Rubisco are sensitive to oxidative modification (Murata et al., 2007; Foyer and Noctor, 2005). Thus, stress protection of photosynthesis depends on the removal of excess ROS, as well as on the maintenance of chloroplast redox homeostasis (Mittler et al., 2011; Murata et al., 2007).

Polyamines (PAs), mainly putrescine (Put), spermidine (Spd), and spermine (Spm), are small aliphatic polycations that accumulate rapidly in plants exposed to unfavorable environments (Alcázar et al., 2020; Blázquez, 2024). Their importance stems from their ability to act at multiple biological levels simultaneously (Roy et al., 2024; Blázquez, 2024). PAs can bind negatively charged membranes, proteins, nucleic acids, and cell wall components; they can influence ion balance and stomatal behavior; and their catabolism through diamine oxidase (DAO; EC 1.4.3.22) and polyamine oxidase (PAO; EC 1.5.3.14) produces H_2_O_2_ that can function as a controlled redox signal (Schomburg and Schomburg, 2005; Tavladoraki et al., 1998; Alcázar et al., 2020). Consequently, recent literature emphasizes that PAs are not merely inert stress metabolites, but are active, multi-functional regulators that link cellular metabolism, stress signaling, development, and defense responses (Alcázar et al., 2020; Blázquez, 2024; Roy et al., 2024).

To organize and synthesize these complex interactions, this review proposes that PA-mediated stress tolerance in fruit crops is governed by a Three-Node Regulatory Axis. The First Node (PA Homeostasis): Comprises stress-regulated PA homeostasis, which is maintained through the coordinated balance of biosynthesis (via enzymes such as arginine decarboxylase [ADC], ornithine decarboxylase [ODC], S-adenosylmethionine decarboxylase [SAMDC], spermidine synthase [SPDS], and spermine synthase [SPMS]) and catabolism. During stress, upregulated PA synthesis maintains the protective cellular PA pool, while controlled catabolism by DAO and PAO generates signaling levels of H_2_O_2_. The Second Node (Antioxidant Activation): Involves antioxidant activation, wherein controlled PA catabolism generates localized H_2_O_2_ signals that prime enzymatic defenses such as superoxide dismutase (SOD), catalase (CAT), ascorbate peroxidase (APX), glutathione reductase (GR), and peroxidase (POD) alongside non-enzymatic antioxidant pools. The Third Node (Photosynthetic Protection): Represents photosynthetic protection, which protects thylakoid membrane stability, preserves PSII photochemical efficiency and chlorophyll pools, regulates stomatal behavior, and supports carbon assimilation.

These three nodes are intimately connected; PA-derived redox signaling activates the antioxidant network, which in turn preserves chloroplast redox balance and maintains photosynthetic carbon fixation under stress. A particularly important unresolved question is whether PAs regulate the expression of Calvin cycle genes in fruit crops. qPCR evidence from salt-stressed *Brassica napus* showed that foliar application of 0.25 mM Spd under 150 mM NaCl stress increased the expression of genes encoding Rubisco subunits, NADP-dependent glyceraldehyde-3-phosphate dehydrogenase (GAPDH; EC 1.2.1.13), fructose-bisphosphate aldolase (FBA; EC 4.1.2.13), sedoheptulose-bisphosphatase (SBPase; EC 3.1.3.37), and fructose-bisphosphatase (FBPase; EC 3.1.3.11), providing a possible link between PA treatment and Calvin cycle regulation (ElSayed et al., 2022). However, comparable RNA-seq, qPCR, promoter, or mutant-based evidence is not yet available for major fruit crops. This missing link is important because maintenance of chlorophyll content may indicate reduced pigment degradation, while an improved F_v_/F_m_ ratio may indicate better maximum photochemical efficiency of PSII; however, neither parameter directly demonstrates that Calvin cycle activity, net CO_2_ assimilation, or carbon fixation are maintained. Therefore, future studies should directly test whether PA treatments regulate Calvin cycle genes and support carbon fixation in fruit crops under abiotic stress.

This review focuses on one mechanistic question: how do PAs regulate antioxidant systems and, in turn, influence photosynthetic performance under abiotic stress, and to what extent can insights from *A. thaliana* be translated into fruit crops? To address this question, the review first summarizes PA biosynthesis, stress-induced accumulation, and catabolism as the biochemical foundation of this regulatory axis. It then examines how PAs modulate enzymatic and non-enzymatic antioxidant defenses and how these responses protect photosynthetic components, including PSII, chlorophyll pools, stomatal function, and carbon fixation. Finally, the review compares the well-characterized molecular framework in *A. thaliana* with the more limited evidence available in fruit crops, highlighting major knowledge gaps and future research priorities. The three figures provide a visual framework for the review, covering polyamine biosynthesis, antioxidant and photosynthetic protection, gaps in knowledge about the Calvin cycle, and the overall PA–antioxidant–photosynthesis model.

## 2 Polyamine biosynthesis and stress-induced accumulation in fruit crops

The amino acids arginine (Arg) and ornithine (Orn) are the principal precursors in plant PA biosynthesis, and both pathways converge to form Put, the central precursor of Spd and Spm. In plants, Put is produced through two routes: the arginine decarboxylase (ADC) pathway and the ornithine decarboxylase (ODC) pathway. In the ADC pathway, Arg is converted into agmatine by ADC (EC 4.1.1.19) (Kusano et al., 2008; Tiburcio et al., 2014). Agmatine is subsequently converted into N-carbamoylputrescine by agmatine iminohydrolase (AIH; EC 3.5.3.12), followed by the hydrolysis of N-carbamoylputrescine to Put by N-carbamoylputrescine amidohydrolase (NCPAH; EC 3.5.1.53) (Kusano et al., 2008; Piotrowski et al., 2003; Tiburcio et al., 2014). In the ODC pathway, Orn is directly converted into Put by ODC (EC 4.1.1.17). S-adenosylmethionine decarboxylase (SAMDC; EC 4.1.1.50) produces decarboxylated S-adenosylmethionine (dcSAM), which donates aminopropyl groups for the conversion of Put into Spd by spermidine synthase (SPDS; EC 2.5.1.16) and Spd into Spm by spermine synthase (SPMS; EC 2.5.1.22) (Kusano et al., 2008; Piotrowski et al., 2003; Schomburg and Schomburg, 2005; Tiburcio et al., 2014).

PA metabolism is also embedded in a broader amino acid and carbon–nitrogen network. Glutamate feeds the biosynthesis of ornithine, arginine, and proline, thereby linking nitrogen assimilation to Put production, while Put oxidation generates 4-aminobutanal that can be converted to γ-aminobutyric acid (GABA) and enter the GABA shunt. Experimental perturbation of Put biosynthesis can alter carbon and nitrogen assimilation and the pools of arginine, ornithine, proline, and GABA, showing that PA accumulation can redistribute metabolic flux rather than acting as an isolated pathway (Majumdar et al., 2016). PA metabolism also intersects with S-adenosylmethionine-dependent ethylene biosynthesis and with ABA and auxin signaling, which is particularly relevant in fruit crops where stress responses overlap with growth, ripening, and source–sink regulation (Bitrián et al., 2012; Mattoo et al., 2006; Tiburcio et al., 2014). Thus, the physiological outcome of PA accumulation depends not only on total PA content but also on the balance among amino acid metabolism, GABA production, hormone signaling, and carbon–nitrogen partitioning.

A key difference between *A. thaliana* and many fruit crops is the presence of the ODC pathway. *A. thaliana* and other Brassicaceae lack ODC and depend on ADC-mediated Put biosynthesis, with ADC2 functioning as the major stress-inducible paralog (Hanfrey et al., 2001; Alcázar et al., 2020). In contrast, several fruit crops, including tomato, citrus, grapevine, apple, and mango, retain both ADC and ODC routes. However, the two pathways do not necessarily contribute equally to Put production, and no general 50:50 relationship has been demonstrated. Their relative contributions vary with the species, organ, developmental stage, nitrogen status, and type and duration of stress. Available evidence suggests that ADC is often the predominant stress-responsive route in fruit crops. For example, salt-induced Put accumulation in apple callus was closely associated with increased *MdADC* expression and ADC activity, whereas ODC activity showed an opposite response (Liu, 2006). Similarly, recent work in *Arabidopsis thaliana* demonstrated that loss of *AtNATA2* markedly enhanced *AtADC2*-dependent putrescine biosynthesis, thereby promoting plant growth and improving salinity tolerance, further highlighting the central role of ADC-mediated putrescine biosynthesis in stress adaptation (Jasso-Robles et al., 2024). Consistent with this, drought stress strongly induced ADC expression in grapevine leaves, while ODC1 and ODC2 were unchanged in the drought-tolerant genotype and only slightly induced in the susceptible genotype (Hatmi et al., 2015). In developing citrus fruit, ADC activity was approximately two- to eightfold higher than ODC activity, although both pathways varied with fruit developmental stage (Nathan et al., 1984). Thus, ADC generally appears to provide the major stress-inducible contribution, whereas ODC may have a greater supplementary, tissue-specific, or developmental role. Nevertheless, pathway dominance should be experimentally determined for each fruit crop rather than assumed. This dual system may provide metabolic flexibility under field stress, but it also means that *Arabidopsis* cannot fully represent PA biosynthesis in fruit crops (Manzoor et al., 2023; Mattoo et al., 2006).

Stress-induced PA accumulation is controlled by hormonal and transcriptional regulation. ABA is the most well-studied signal that can trigger ADC2 expression and Put buildup in *Arabidopsis*. JA and other stress-related signals can also influence genes involved in PA biosynthesis and catabolism (Alcázar et al., 2020). In grapevine, ABA stimulates ADC, ODC, and SAMDC activities and promotes PA export to the apoplast, linking PA metabolism with cell wall-associated and apoplastic redox responses under stress (Toumi et al., 2010). However, direct evidence identifying the transcription factors that bind ADC, ODC, SAMDC, and PAO promoters remains limited in fruit crops.

Polyamines (PAs) catabolism is also critical because DAO primarily oxidizes Put, whereas PAOs oxidize Spd and Spm, generating H_2_O_2_. At high concentrations, H_2_O_2_ can damage cells, but at controlled concentrations, it functions as a redox signal. Recent evidence has advanced this concept beyond a general priming hypothesis. In tomatoes, treatment with 0.25 mM Spd increased *SlPAO4* expression by 3.69-fold within 1 h, followed by increased PAO activity and H_2_O_2_ production. Inhibition of PAO activity or silencing of *SlPAO4* reduced antioxidant activation and weakened saline–alkaline stress tolerance, demonstrating a direct role of PAO-derived H_2_O_2_ in stress signaling (Yang et al., 2022). The chloroplast connection is supported by the localization of ADC in thylakoid-associated chloroplast fractions and the detection of a substantial Put pool in plastids (Borrell et al., 1995; Krueger et al., 2011). However, a critical biochemical distinction must be made between chloroplast-associated Put generation and the biosynthesis of higher polyamines. While ADC-mediated Put synthesis is structurally linked to chloroplast thylakoids, direct protein-level evidence showing that the downstream aminopropyltransferases spermidine synthase (SPDS) and spermine synthase (SPMS) are localized or active within chloroplasts is completely lacking in fruit crops. Consequently, complete chloroplastic biosynthesis of Spd and Spm remains a hypothetical model rather than an established fact. Furthermore, although some polyamine catabolic activity has been reported in isolated plastids, convincing protein-level evidence for the localization of a specific functional DAO or PAO enzyme inside higher-plant chloroplasts remains extremely limited, with virtually no direct validation in fruit crop species (Andreadakis and Kotzabasis, 1996).

Regulation of PA catabolism also occurs post-translationally. In woodland strawberry, FvCSN5A interacts with FvPAO5 and facilitates its ubiquitination and degradation, thereby altering PA and H_2_O_2_ homeostasis, plant development, fertility, and fruit ripening (Huang et al., 2025). Although this study was not conducted under abiotic stress, it demonstrates that PAO abundance can be controlled by protein turnover, adding a mechanistic layer that may determine the strength and duration of PA-derived H_2_O_2_ signaling.

The subcellular location of PA metabolism is also important for interpreting the pathway in **Figure 1**. Both chloroplasts and mitochondria are major sites of stress-induced ROS production because over-reduction of their electron-transport chains can increase ROS formation under drought, salinity, and heat stress (Ugalde et al., 2021). Because ADC is associated with chloroplast thylakoids and Put is present in plastids, local PA accumulation may rapidly influence PSII stability, thylakoid charge balance, and stromal redox status (Borrell et al., 1995; Krueger et al., 2011). However, because SPDS and SPMS have not been directly localized to chloroplasts, the presence of local Spd and Spm pools within the plastid likely relies on cytosolic synthesis followed by active envelope transport, rather than complete *in situ* chloroplastic biosynthesis. In fruit crops, experimental evidence localizing PA-metabolic enzymes specifically to chloroplasts or mitochondria remains limited. Most available studies have focused on gene identification and expression or have identified PA-catabolic enzymes in other compartments, such as the peroxisomal *MdAO1* and extracellular *MdAO2* in apple; therefore, the proposed chloroplast and mitochondrial roles are still based largely on speculative extrapolation from *Arabidopsis* and other model or non-fruit plant species (Sadiq et al., 2023; Tavladoraki et al., 1998; Wang et al., 2019; Zarei et al., 2015). Direct evidence from organelle fractionation, immunoblotting, GFP-tagged proteins, or immunolocalization would strongly improve the mechanistic interpretation of **Figure 1**.

**Figure 1:**
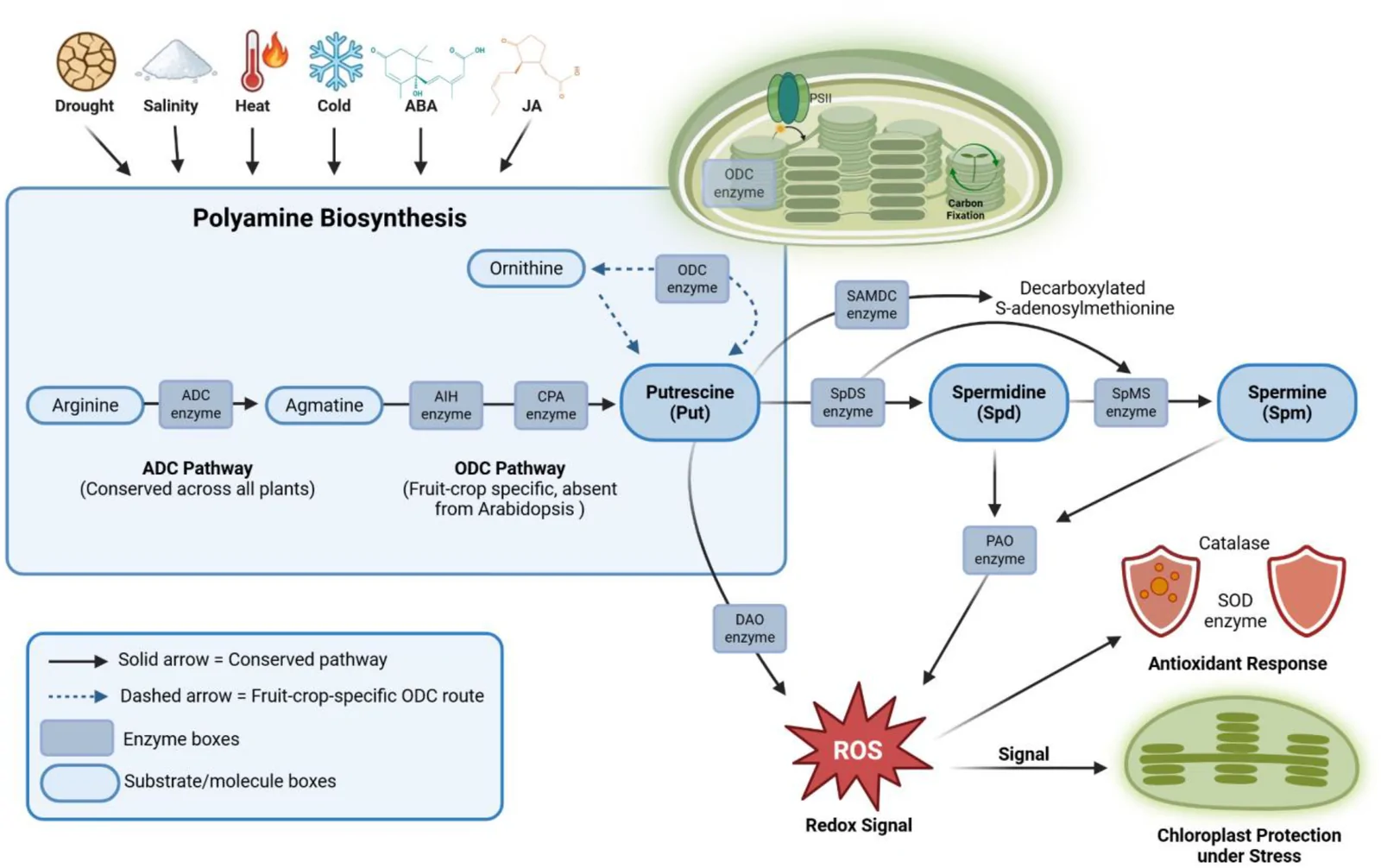
Polyamine biosynthesis, stress-induced regulation, and catabolism in fruit plants. The conserved arginine decarboxylase (ADC) pathway converts arginine to agmatine, then to putrescine via AIH and NCPAH. The ornithine decarboxylase (ODC) pathway directly converts ornithine to putrescine and is present in many fruit crops but absent from *Arabidopsis* and other Brassicaceae. Putrescine is converted to spermidine and spermine through SpDS and SpMS using dcSAM supplied by SAMDC. PA catabolism through DAO and PAO generates H_2_O_2_, which can function as a redox signal for antioxidant activation when maintained at controlled levels. Created in BioRender. Jan, M. (2026) https://BioRender.com/z86orag.

Species-specific evidence supports the presence of this biosynthetic response and shows that fruit crops differ from *Arabidopsis* in important biological and regulatory features. In apple callus exposed to 200 mM NaCl, free Put was approximately 180% higher than the control after 1 day and 74% higher after 3 days. This accumulation was associated with increased *MdADC* expression and ADC activity, whereas ODC activity did not show a corresponding increase (Liu, 2006). Grapevine studies further show that ABA can activate ADC, ODC, and SAMDC and promote PA movement toward the apoplast (Toumi et al., 2010). However, the magnitude of PA accumulation varies considerably across species, tissues, PA forms, stress intensities, and sampling times. Therefore, quantitative values should be reported for individual experiments rather than presented as a single general percentage for all fruit crops. These examples support the central pathway in **Figure 1**, but the regulatory details, including which transcription factors activate ADC, ODC, SAMDC, SpDS, and SpMS in fruit crops, remain poorly defined.

## 3 Polyamine-mediated regulation of the antioxidant defense system

The antioxidant defense system forms the central link between PA accumulation and photosynthetic protection. PA treatment or the natural accumulation of PAs under stress is frequently associated with increased activity of key antioxidant enzymes, including SOD, CAT, APX, GR, and POD. For example, exogenous Put enhanced the activities of SOD, CAT, APX, and GR and improved salt tolerance in tomato seedlings under NaCl stress, while exogenous Spd enhanced SOD, CAT, APX, and GR activities and improved the chloroplast antioxidant system in tomato seedlings exposed to saline-alkaline stress (Li et al., 2015; Yang et al., 2022; Rao et al., 2025). These enzymes work together to control reactive oxygen species. SOD converts superoxide into H_2_O_2_, while CAT and APX detoxify H_2_O_2_ and prevent its harmful buildup. GR helps maintain reduced glutathione levels, which are essential for redox balance, and POD contributes to H_2_O_2_ metabolism in the apoplast. Together, these antioxidant responses reduce lipid peroxidation, limit membrane leakage, and help protect chloroplast function under stress (Das and Roychoudhury, 2014; Gill and Tuteja, 2010; Li et al., 2015). The ascorbate-glutathione cycle is especially important in chloroplasts because it detoxifies H_2_O_2_ while maintaining reduced ascorbate and glutathione pools. APX uses ascorbate to reduce H_2_O_2_ to water. Monodehydroascorbate reductase (MDHAR) and dehydroascorbate reductase (DHAR) regenerate ascorbate, while GR regenerates reduced glutathione using NADPH. PA-treated plants often show higher APX and GR activities and improved AsA/DHA and GSH/GSSG balance, suggesting that PAs help preserve chloroplast redox poise under stress (Foyer and Noctor, 2005; Li et al., 2015; Yang et al., 2022).

Polyamines also influence non-enzymatic antioxidants, including ascorbate, glutathione, proline, phenolics, flavonoids, carotenoids, and tocopherols. These metabolites provide additional ROS scavenging, osmotic adjustment, and membrane protection. In fruit crops, PA-associated increases in phenolic concentrations and antioxidant capacity are particularly relevant because these compounds influence both stress tolerance and fruit quality (Manzoor et al., 2023). However, most studies measure antioxidant activity or metabolite pools rather than the gene-regulatory mechanisms that control these responses. A key feature of this system is the dual role of H_2_O_2_. Polyamine catabolism produces not only H_2_O_2_ but also several nitrogen-containing metabolites with potential physiological functions. DAO-mediated oxidation of Put produces 4-aminobutanal, ammonia, and H_2_O_2_. The 4-aminobutanal can subsequently be converted into γ-aminobutyric acid (GABA), linking PA catabolism with stress signaling, carbon-nitrogen metabolism, and the mitochondrial GABA shunt. Terminal oxidation of Spd and Spm by PAO produces 1,3-diaminopropane and aminoaldehydes, along with H_2_O_2_, whereas PAO-mediated back-conversion regenerates Spd or Put, thereby contributing to the maintenance of cellular PA homeostasis. However, the specific stress-related functions of 1,3-diaminopropane and the aminoaldehyde products remain less clearly defined than those of H_2_O_2_ and GABA (Wang et al., 2019).

A strong oxidative burst can damage membranes, proteins, and enzymes, whereas a small and localized H_2_O_2_ signal can activate defense responses before stress becomes severe. Stress-induced export and oxidation of Spd in the apoplast have been shown to generate H_2_O_2_ signatures that regulate tolerance responses, while tomato studies demonstrate that Spd-induced PAO activity and H_2_O_2_ production activate downstream antioxidant defenses (Moschou et al., 2008; Yang et al., 2022). In this context, PA catabolism may function as a redox switch. DAO-derived H_2_O_2_ in the apoplast can promote calcium influx, MAPK signaling, and the activation of stress-responsive transcription factors. At the same time, PAO-dependent back-conversion can help maintain PA balance within the cell. As shown in **Figure 2**, this route connects the catabolic branch of PA metabolism (**Figure 1**) to antioxidant enzyme activation and the AsA–GSH cycle, thereby providing a mechanistic link between PA metabolism and stress protection.

**Figure 2:**
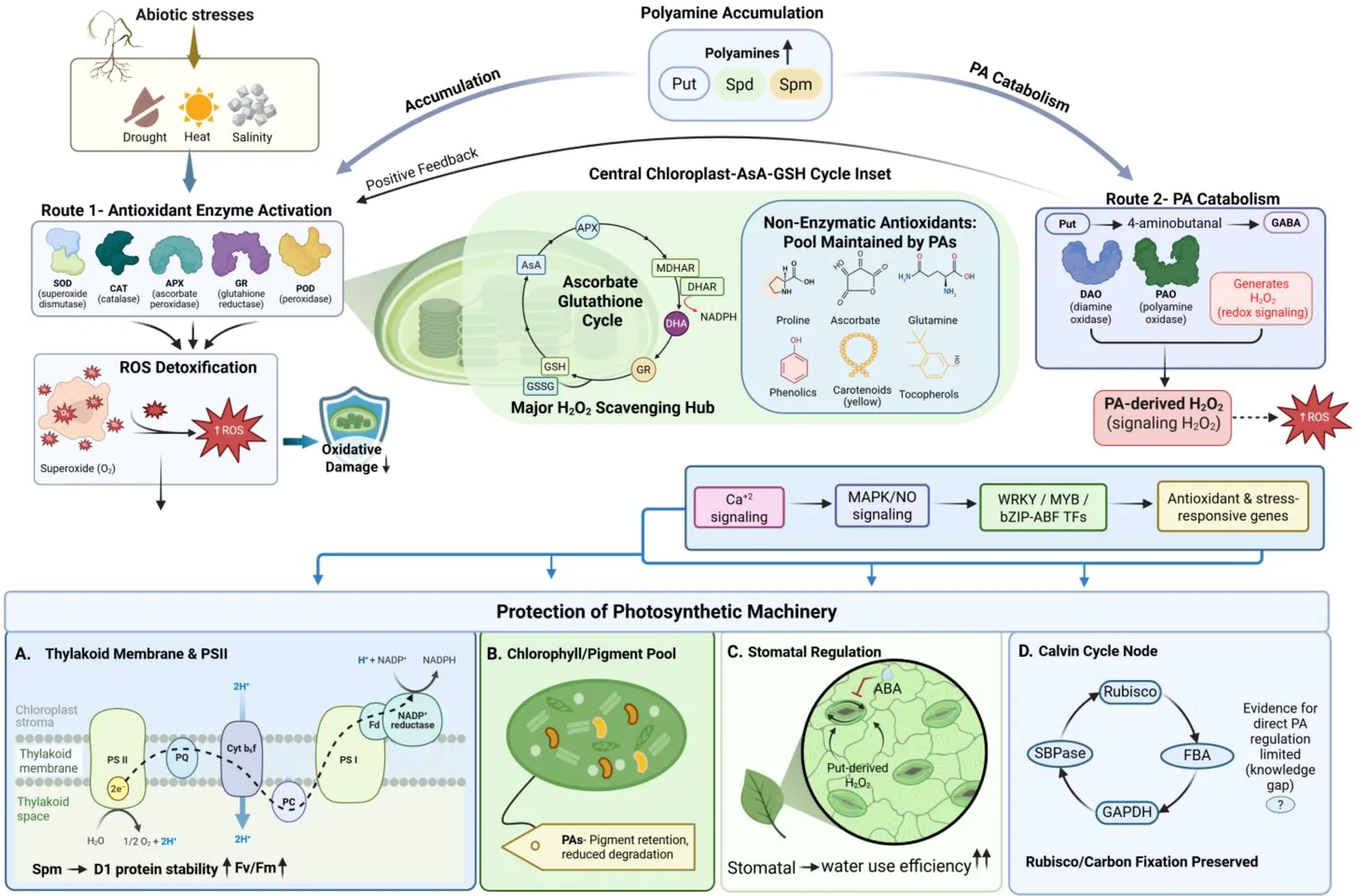
Polyamine-mediated antioxidant defense and protection of photosynthetic machinery under abiotic stress. Abiotic stresses promote polyamine (PA) accumulation and activate enzymatic and non-enzymatic antioxidant defenses, including the chloroplast ascorbate–glutathione cycle. PA catabolism through DAO and PAO generates H_2_O_2_, which at controlled levels functions as a redox signal, while Put catabolism also connects PA metabolism with GABA formation. PA-derived H_2_O_2_ can activate Ca²^+^-, MAPK-, and NO-associated signaling and stress-responsive transcriptional regulators, contributing to antioxidant gene activation and stress adaptation. Together, these responses limit oxidative injury, stabilize thylakoid membranes and PSII, preserve chlorophyll and accessory pigments, and influence ABA-associated stomatal regulation. Direct PA regulation of Calvin-cycle components in fruit crops remains insufficiently established and is highlighted as a major knowledge gap. Created in BioRender. Jan, M. (2026) https://BioRender.com/axcm0oo.

Beyond antioxidant activation, PA signals can engage canonical second-messenger and protein-kinase pathways. In tobacco, Spm-dependent activation of the SIPK/WIPK MAPKs required ROS production and Ca²^+^ influx (Takahashi et al., 2003), while in *Arabidopsis*, Spm-induced unfolded-protein-response signaling depended on Ca²^+^ influx and the MKK9–MPK3/MPK6 cascade (Sagor et al., 2015). PAs also interact with nitric oxide (NO) signaling; exogenous Put, Spd, and Spm induced NO production in Arabidopsis, supporting NO as one mediator of PA-dependent signaling (Wimalasekera et al., 2011). Importantly, recent tomato evidence links PA transport directly to downstream stress signaling: SlPUT3 interacted with the H_2_O_2_ transporter SlPIP2;4, and Spm induced SlMAPK3 and several stress-responsive genes in an H_2_O_2_-dependent manner (Yang et al., 2024). Together, these findings support a working sequence in which PA transport or catabolism modifies H_2_O_2_/ROS and Ca²^+^/NO signals, activates MAPK and other Ca²^+^-dependent signaling modules, and changes downstream stress-responsive gene expression. The conservation and relative importance of these signaling branches remain incompletely resolved in perennial fruit crops.

Fruit crop studies generally measure antioxidant activities rather than the transcriptional events behind them. This means that increased SOD, CAT, APX, GR, or POD activity after PA treatment is well documented, but the underlying gene regulatory mechanism remains unclear. For example, it is not yet known whether PA treatment directly activates antioxidant promoters, whether PA-derived H_2_O_2_ activates a specific transcription factor (TF) module, or whether the response is secondary to reduced stress injury. Future studies should combine enzyme activity assays with transcript abundance, promoter analysis, and inhibitor or mutant approaches to test the antioxidant response in **Figure 2** as a causal mechanism rather than merely a physiological association.

## 4 Polyamine-dependent protection of photosynthetic components

The antioxidant capacity induced by PAs protects several photosynthetic targets, including thylakoid membranes, PSII, chlorophyll and accessory pigments, stomatal regulation, and Calvin cycle activity. These targets are connected, but they are not the same. Some PA effects are structural, such as stabilization of the thylakoid membrane and protection of PSII. Others are biochemical, such as maintaining the AsA-GSH cycle and preserving Rubisco activity (Li et al., 2015; ElSayed et al., 2022). Other effects involve regulation, such as how H_2_O_2_ from PAs helps with signaling in guard cells and controlling stomata. As shown in **Figure 2**, these different layers of protection work together to maintain photosynthetic performance under abiotic stress.

Spermine (Spm) is particularly effective at protecting thylakoid membranes because of its high positive charge. Its polycationic nature enables electrostatic interactions with negatively charged components of the thylakoid and PSII, which can stabilize PSII under photoinhibitory conditions (Hamdani et al., 2011). This protection can help preserve PSII photochemical function. However, the identity and concentration of the applied PA, as well as the type of stress, should be specified for each study rather than combining experiments into a single general statement. In ‘Red Tangerine’ citrus *in vitro* plants, pretreatment with 1 mM Spm before dehydration reduced wilting, water loss, and electrolyte leakage. It also increased endogenous PA levels and the expression of ADC and SPMS, enhanced SOD and POD activities, reduced ROS accumulation, and promoted stomatal closure (Shi et al., 2010). These results show antioxidant and stomatal protection during dehydration, although that study did not directly measure Fv/Fm or PSII.

This effect likely reflects reduced oxidative damage to pigment–protein complexes, improved thylakoid stability, and possible effects on chlorophyll catabolic enzymes. However, the citrus study by Shi et al. (2010) did not quantify chlorophyll content or the activities or expression of chlorophyll catabolic enzymes. Therefore, it should not be used as direct evidence for reduced chlorophyll degradation. Direct regulation of chlorophyllase or other chlorophyll catabolic enzymes by PAs in fruit crops remains insufficiently demonstrated.

Stomatal regulation provides another route by which PAs support photosynthesis under water-deficit stress. Put is the specific PA involved in the DAO-mediated reaction. Its oxidation produces 4-aminobutanal, NH_3_>, and H_2_O_2_, and 4-aminobutanal can subsequently be converted into GABA, thereby connecting Put catabolism with redox signaling and carbon–nitrogen metabolism (Wang et al., 2019). The resulting H_2_O_2_ can participate in ABA-associated guard-cell signaling and stomatal closure. In citrus, pretreatment with 1 mM Spm increased endogenous PA accumulation and ADC and SPMS expression, strengthened SOD- and POD-dependent ROS scavenging, and reduced stomatal aperture and water loss during dehydration (Shi et al., 2010). These responses indicate that PA-mediated protection involves both the regulation of stomatal limitation, through control of stomatal aperture and transpiration, and the alleviation of non-stomatal limitations, through improved antioxidant protection and maintenance of cellular metabolism.

The least understood target is the Calvin cycle. In salt-stressed *Brassica napus*, treatment with 0.25 mM Spd under 150 mM NaCl increased chlorophyll content, PSII activity, CO_2_ assimilation, Rubisco activity, and the qRT-PCR-measured expression of genes encoding Rubisco subunits, GAPDH, PGK, TPI, FBA, SBPase, and FBPase. The same concentration of Spm was also examined, but Spd generally provided stronger protection (ElSayed et al., 2022). Therefore, this was a qRT-PCR study under salinity stress, rather than a transcriptomic study under drought stress. This finding provides a direct molecular link between PA treatment and the regulation of carbon-fixation genes. In citrus, Shi et al. (2010) showed that Spm pretreatment enhanced dehydration tolerance through improved antioxidant capacity, reduced oxidative damage, and modulation of stomatal responses, but Calvin-cycle gene regulation was not examined. However, similar evidence from RNA-seq, qPCR, promoter analysis, mutant studies, or biochemical validation has not yet been reported in fruit crops. This represents one of the most important knowledge gaps in the field because it directly connects PA treatment with the maintenance of carbon assimilation and yield under stress.

Although PA treatments have frequently been reported to maintain chlorophyll content, Fv/Fm, and photosynthetic rate under stress, these responses do not necessarily demonstrate direct regulation of carbon assimilation by PAs. Fv/Fm is a dark-adapted estimate of the maximum photochemical efficiency of PSII. Therefore, maintenance of Fv/Fm following PA treatment indicates that PSII has experienced less photoinhibition, but it does not directly demonstrate that net CO_2_ assimilation is maintained. Carbon assimilation can remain restricted because of stomatal closure, reduced Rubisco activation, limited RuBP regeneration, or impaired triose-phosphate metabolism, even when Fv/Fm and chlorophyll content remain relatively stable (Maxwell and Johnson, 2000). Although Spd treatment has been associated with improved photosynthetic performance and CO_2_ assimilation in stressed tomato plants, the underlying effects on Calvin-cycle regulation remain insufficiently characterized. In fruit crops, this is directly relevant to yield because sustained CO_2_ fixation supports fruit growth, sugar accumulation, and source–sink balance. Therefore, the Calvin-cycle node shown in **Figure 2** should be treated as a central research target for understanding PA-mediated protection of photosynthesis rather than as a minor downstream endpoint.

A possible connection between PAs and Rubisco activase also requires further investigation. Changes in Rubisco activase abundance after PA treatment should be interpreted with caution, as greater protein abundance does not necessarily indicate higher Rubisco activation (Salvucci et al., 2001). Rubisco activase is an ATP-dependent molecular chaperone, and its activity is regulated by light-dependent changes in the stromal ATP/ADP ratio and redox state (Zhang and Portis, 1999). It is also particularly sensitive to heat. Therefore, studies examining the effects of PAs on Rubisco activase abundance should also assess irradiance, Rubisco activase activity, Rubisco activation state, and gas-exchange measurements (Portis, 2003; Zhang and Portis, 1999). Although PAs have been shown to improve photosynthetic performance under stress, direct evidence that PAs regulate Rubisco activase or the expression and activity of Calvin-cycle enzymes in fruit crops remains limited. Thus, a key experiment remains missing: PA treatment, stress exposure, photosynthetic measurements, and qPCR or RNA-seq validation of Calvin-cycle genes within the same experimental design. This missing evidence is the focus of **Figure 3**.

**Figure 3:**
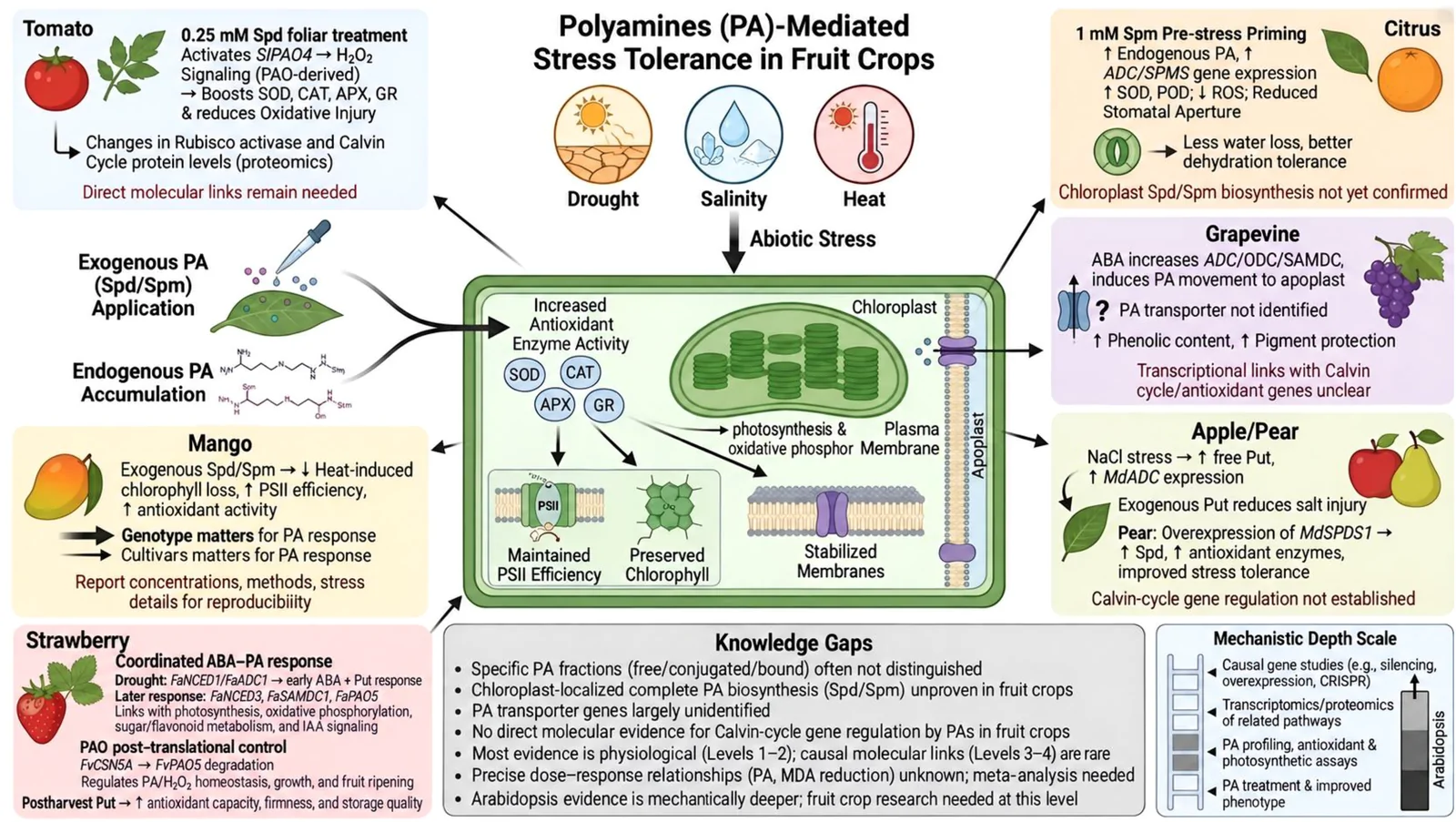
Polyamine-mediated regulation of abiotic stress tolerance in fruit crops and major knowledge gaps. Exogenous PA application and stress-induced endogenous PA accumulation can enhance antioxidant activity, membrane stability, chlorophyll retention, PSII performance, stomatal regulation, and stress tolerance in fruit crops. Representative evidence from tomato, citrus, grapevine, apple/pear, mango, and strawberry illustrates species-specific PA responses under drought, salinity, heat, and related stresses. Strawberry studies additionally demonstrate coordinated ABA–PA regulation during drought and post-translational control of PAO5, extending current evidence beyond postharvest responses. Despite these advances, PA transport, organelle-specific PA metabolism, direct regulation of Calvin-cycle genes, and causal downstream signaling remain poorly resolved in many fruit crops. The mechanistic-depth scale emphasizes the need to move from physiological associations toward transcriptomic, proteomic, genetic, and functional validation. Created in BioRender. Jan, M. (2026) https://BioRender.com/grv1lpq.

## 5 *Arabidopsis* as a molecular reference framework

*Arabidopsis thaliana* provides the strongest molecular framework for understanding the PA stress response. In this model species, Put biosynthesis depends on ADC1 and ADC2, and both genes contribute to cold-induced Put accumulation and freezing tolerance (Cuevas et al., 2008). Constitutive ADC2 overexpression markedly increases Put accumulation but also causes developmental effects, including dwarfism and delayed flowering through altered gibberellin metabolism (Alcázar et al., 2005). Most of these studies primarily quantified the free Put pool, whereas conjugated, bound, and acetylated PA fractions were examined less consistently. Therefore, changes in total PAs should not be interpreted without identifying the specific PAs and their metabolic fraction. Recent evidence further shows that PA turnover contributes to stress adaptation: loss of *AtNATA2* enhances *AtADC2*-dependent Put biosynthesis and stress priming, resulting in improved growth and salinity tolerance (Jasso-Robles et al., 2024). This finding indicates that free and acetylated Put pools are metabolically connected and that PA homeostasis, rather than total PA concentration alone, contributes to stress protection.

Transgenic evidence further supports the importance of Spd. Overexpression of SPDS increases Spd accumulation and enhances tolerance to chilling, freezing, salinity, hyperosmotic, drought, and oxidative stresses, accompanied by upregulation of several stress-responsive genes (Kasukabe et al., 2004). Urano et al. (2003) independently demonstrated stress-dependent regulation of endogenous PA-biosynthetic genes in *Arabidopsis*. Direct evidence linking PA treatment with Calvin-cycle gene expression instead comes from salt-stressed *Brassica napus* (ElSayed et al., 2022). Where individual PA fractions were measured, the response was mainly associated with increased free Spd; however, conjugated, bound, and acetylated fractions were not consistently quantified and should be specified when interpreting these studies. Together with the recent *AtNATA2* study, these findings demonstrate that both PA biosynthesis and subsequent metabolic conversion influence stress tolerance. These findings provide testable hypotheses for fruit crops, where equivalent molecular evidence remains limited.

The *A. thaliana* framework also includes strong ABA–PA crosstalk. Stress-responsive PA-biosynthetic genes, particularly ADC2, are transcriptionally regulated by ABA and abiotic stress, while cold-induced Put accumulation can influence ABA metabolism and signaling (Urano et al., 2003; Cuevas et al., 2008; Alcázar et al., 2020). Direct promoter binding by specific transcription factors to PA-metabolic genes, however, remains incompletely established. However, direct extrapolation from *A. thaliana* to fruit crops has limits. *A. thaliana* lacks the ODC pathway and produces dry, dehiscent siliques rather than fleshy fruits (Hanfrey et al., 2001). Although it has a clearly defined fruit-development program, it does not reproduce the fleshy-fruit expansion, ripening physiology, prolonged source-sink relationships, or perennial woody growth of most fruit crops. Fruit crops also have fruit-specific PA ethylene interactions, longer stress exposure under field conditions, and more complex developmental sinks. Therefore, *Arabidopsis* should be used as a hypothesis-generating framework, not as a substitute for fruit-crop molecular studies.

The value of *A. thaliana* is therefore not that it provides a complete explanation for fruit crops but that it provides testable hypotheses. ADC2-centered Put biosynthesis, PAO-mediated H_2_O_2_ signaling, ABA–PA crosstalk, and WRKY-, MYB-, or bZIP-linked promoter regulation can be used as starting points for fruit-crop experiments. The important step is to test whether these modules are conserved, modified, or replaced by crop-specific networks. This is particularly relevant for species that retain the ODC pathway because stress-induced Put accumulation may be distributed between ADC and ODC in ways that *Arabidopsis* cannot model (Alcázar et al., 2005; Hanfrey et al., 2001; Manzoor et al., 2023).

Another important limitation is developmental context. Fruit crops contain vegetative tissues, reproductive organs, and developing fruits, each of which may differ in PA metabolism, ethylene sensitivity, antioxidant capacity, and sink demand (Aucique-Pérez et al., 2026; Tiburcio et al., 2014). During fruit ripening, PAs can interact with ethylene and influence firmness, senescence, and postharvest quality. In leaves, PAs are more closely linked with redox balance, antioxidant defense, and photosynthetic protection (Alcázar et al., 2020; Blázquez, 2024). Therefore, future studies in fruit crops should clearly define the tissue under examination and avoid conflating leaf-based photosynthetic mechanisms with postharvest fruit-quality responses unless this connection is directly tested.

## 6 Fruit crops: current knowledge and major gaps

Tomato is the most extensively studied fruit crop for PA-mediated stress tolerance. Heat and cold stress differentially alter endogenous PA pools and the expression of PA biosynthesis and catabolism genes (Upadhyay et al., 2020). Separately, exogenous Spd or Spm can enhance antioxidant and photosynthetic protection under saline–alkaline, combined salinity/oxidative, and heat stress conditions (Yang et al., 2022; Pascual et al., 2023; Sang et al., 2017). The PA fraction should be specified because stress and exogenous treatments can affect free, conjugated, and bound pools differently. Recent mechanistic evidence showed that foliar pretreatment with 0.25 mM Spd activated SlPAO4, increased PAO-derived H_2_O_2_ signaling, enhanced SOD, CAT, APX, and GR responses, and reduced oxidative injury in tomato seedlings exposed to saline–alkaline stress (Yang et al., 2022). Proteomic evidence in heat-stressed tomato indicates that Spd can alter the abundance of chloroplast-associated antioxidant and photosynthetic proteins, including Rubisco activase and Calvin-cycle-related proteins (Sang et al., 2017). While this physiological evidence is promising, establishing direct molecular links will strengthen confidence in the role of PAs in stress tolerance. More recently, SlPUT3-mediated PA transport was linked to H_2_O_2_ signaling through interaction with SlPIP2;4; Spm induced SlMAPK3 and several stress-responsive genes, and this induction was weakened by H_2_O_2_ scavenging, providing direct evidence for a PA/H_2_O_2_-to-MAPK signaling bridge in tomato (Yang et al., 2024).

Citrus provides strong physiological evidence for PA-mediated protection (Aucique-Pérez et al., 2026; Shi et al., 2010). Pretreatment with 1 mM Spm before dehydration should be described as pre-stress priming rather than stimulation during stress. This treatment increased endogenous PA accumulation and the expression of ADC- and SPMS-related genes, enhanced SOD and POD activities, reduced ROS accumulation, and decreased stomatal aperture. The smaller stomatal aperture limited transpiration water loss and contributed to improved tissue water status during dehydration (Shi et al., 2010). This makes citrus a valuable system for testing the complete PA–antioxidant–photosynthesis axis. Regarding chloroplast PA metabolism, while model system evidence supports chloroplast-localized ADC and subsequent local Put production, direct protein-level evidence showing that SPDS or SPMS resides inside citrus chloroplasts has not yet been established. Thus, complete Spd or Spm biosynthesis within the chloroplast must be treated strictly as a hypothesis, with plastidal Spd and Spm accumulation likely depending on transport from the cytosol (Borrell et al., 1995). Upstream transcription factors, the organelle-specific functions of the PAO gene family, and direct downstream responses of Calvin cycle genes remain completely unresolved in citrus. Addressing these specific gaps is essential to validate these proposed subcellular mechanisms.

A complementary regulatory mechanism has been established in the closely related trifoliate orange (*Poncirus trifoliata*). Under drought conditions, PtrSnRK2.4 phosphorylates PtrABF2, and phosphorylated PtrABF2 directly binds to the PtrADC promoter to enhance ADC expression, Put accumulation, and drought tolerance (Song et al., 2023). This SnRK2.4–ABF2–ADC module provides a concrete example of how canonical ABA signaling can be connected to PA biosynthesis in citrus-related germplasm and offers a testable framework for cultivated Citrus species.

Grapevine is especially useful for studying ABA–PA interactions. ABA can change how the plant uses PA by boosting the activities of ADC, ODC, and SAMDC and helping PA move toward the apoplast (Toumi et al., 2010). This redistribution requires membrane transport, because PA transporters regulate cellular uptake, efflux, long-distance movement, and subcellular compartmentation. However, the original study did not identify the transporter responsible for ABA-associated PA export into the grapevine apoplast, and functionally validated PA transporters remain poorly characterized in grapevines. Notably, recent work in tomato functionally identified PUT2 as a regulator of shoot-to-root PA transport and demonstrated that the HD-Zip transcription factor HB52 directly activates PUT2 during salt stress (Yang et al., 2025). Thus, PA transport is no longer entirely uncharacterized in fruit crops, although transporter identity, substrate specificity, regulation, and subcellular functions remain poorly resolved across most fruit species. Within grapevine, the strongest direct evidence in this system supports ABA-dependent reorientation of PA metabolism and apoplastic PA/H_2_O_2_ signaling (Toumi et al., 2010). However, transcriptional links among PA metabolism, antioxidant genes, and Calvin-cycle genes remain poorly characterized.

Apples and pears provide evidence from both tissue-culture and transgenic systems. In apple callus, the PA response to 200 mM NaCl was primarily endogenous: salt stress increased free Put throughout the treatment period and transiently increased conjugated Put during the early response. These changes were associated with increased MdADC expression and ADC activity, whereas ODC activity did not increase correspondingly. The same study showed that exogenous Put application could alleviate salt injury (Liu, 2006). In pear, overexpression of MdSPDS1 increases Spd levels and improves tolerance to salinity and osmotic stress, with higher antioxidant enzyme activities and reduced oxidative damage (Wen et al., 2008; He et al., 2008). This provides valuable genetic evidence in a fruit-tree system, but it still does not close the Calvin-cycle gene-regulation gap.

In mango, the strongest direct evidence for exogenous PA application concerns postharvest fruit quality rather than the heat protection of the photosynthetic apparatus. Exogenous Spm, Spd, or Put treatments have been reported to delay softening and color development, reduce weight loss and deterioration, and maintain firmness and other quality attributes during storage; in ‘Langra’ mango, 2 mM Spd was among the most effective treatments for maintaining firmness and delaying deterioration (Malik et al., 2006; Zahedi et al., 2019). These postharvest findings should not be interpreted as direct evidence that PAs protect PSII, chlorophyll, or gas exchange during heat stress in mango plants.

Strawberry now provides both stress-response and postharvest evidence. Under drought, Gao et al. (2024) showed temporal coordination between Put and ABA: early drought promoted FaADC1-associated Put accumulation and FaNCED1-associated ABA accumulation, whereas later ABA accumulation reprogrammed PA homeostasis by suppressing FaADC1 and promoting FaSAMDC1 and FaPAO5. RNA-seq and physiological analyses linked Put with photosynthesis and oxidative phosphorylation and linked ABA with sugar and flavonoid metabolism, with IAA-associated crosstalk proposed between these responses. This study extends strawberry PA research beyond harvested fruit and demonstrates a molecular hormone–PA network during drought. Separately, postharvest immersion in Put (0.3–2.0 mM) reduced ethylene production and fruit softening, maintained firmness and marketable quality, and extended storage life, with 1.0 and 2.0 mM treatments showing the strongest effects (Khosroshahi et al., 2007). At the post-translational level, FvCSN5A was recently shown to interact with FvPAO5 and facilitate its degradation, thereby regulating PA/H_2_O_2_ homeostasis, plant growth, fertility, and fruit ripening (Huang et al., 2025).

Across fruit crops, PA treatments or increases in endogenous PAs are frequently associated with greater antioxidant capacity, lower oxidative damage, smaller stress-induced losses of chlorophyll, and improved photosynthetic indicators. However, the available literature does not establish a universal quantitative relationship between the percentage increase in PAs and the percentage reduction in MDA. Studies differ in PA identity, free or conjugated fraction, treatment concentration, tissue, stress intensity, treatment duration, sampling time, and analytical method. Therefore, an equivalent percentage increase in PA content should not be expected to produce the same reduction in MDA across different species or experiments.

A formal meta-analysis would be required to quantify this relationship. PA accumulation and MDA reduction should first be expressed as percentage changes relative to the corresponding stressed control. PA identity, metabolic fraction, treatment concentration, crop species, tissue, stress type, stress severity, and sampling time could then be evaluated as moderator variables. This approach could identify the effective range of PA accumulation, determine whether a response threshold or saturation point exists, and distinguish protective PA concentrations from levels that may stimulate excessive PA oxidation and H_2_O_2_ production.

The current fruit-crop literature therefore provides repeated physiological associations rather than uniform mechanistic validation. Studies on tomato, citrus, grapevine, apple, pear, mango, and strawberry report combinations of lower MDA or electrolyte leakage, increased antioxidant enzyme activity, reduced chlorophyll loss, and improved chlorophyll fluorescence or gas-exchange parameters. However, most studies use endpoint measurements and do not determine whether PA accumulation precedes antioxidant activation, whether PA-derived H_2_O_2_ is required, or whether photosynthetic recovery depends on regulation of Calvin-cycle genes.

An important next step would be to classify the available fruit-crop evidence according to mechanistic depth. Level 1 evidence includes PA treatment and an improved stress phenotype. Level 2 includes PA profiling, antioxidant assays, and photosynthetic measurements. Level 3 includes transcriptomic or proteomic evidence for antioxidant- and photosynthesis-related pathways. Level 4 includes causal validation through gene silencing, overexpression, CRISPR, promoter assays, or pharmacological inhibition of DAO or PAO. Most fruit-crop studies currently fall between Levels 1 and 2, whereas *Arabidopsis* provides more Level 3 and Level 4 evidence. This imbalance explains why direct molecular validation in fruit crops remains the main priority and why **Figure 3** is positioned as the primary knowledge gap.

## 7 Exogenous polyamine application and photosynthetic recovery in fruit crops

Exogenous PA applications have practical value because they can trigger protective responses before or during stress. Researchers have used foliar spray, root application, seed priming, and postharvest dipping, depending on the crop and stress context. Foliar applications support rapid leaf responses, while root applications may better influence osmotic adjustment and whole-plant water relations. Seed or plant priming with small-molecule biostimulants can enhance stress preparedness, although seed priming is less directly relevant to perennial fruit trees than to annual horticultural crops (Ugena et al., 2018; Hernándiz et al., 2022).

Application timing is critical for PA effectiveness. Pretreatment can be advantageous because it activates antioxidant defenses and redox priming before severe oxidative injury develops. This concept is consistent with the broader use of small molecule-based biostimulants, including polyamines, where seed or plant priming enhances stress preparedness and improves subsequent tolerance to abiotic stress (Ugena et al., 2018; Hernándiz et al., 2022). Once severe oxidative damage has affected membranes, chlorophyll pools, thylakoid membranes, and PSII function, full photosynthetic recovery becomes less likely because oxidative stress can inhibit PSII repair and intensify photoinhibition (Nishiyama et al., 2001; Murata et al., 2007). Accordingly, PA treatments may be most effective as preventive or early-intervention biostimulants rather than as rescue treatments (Ugena et al., 2018; Hernándiz et al., 2022).

The dose and type of PAs also influence the response. Put may be more closely associated with DAO-derived H_2_O_2_ signaling and stomatal regulation, whereas Spd and Spm, because of their higher positive charge density, may provide stronger protection of membranes and thylakoid structures (Alcázar et al., 2020; Hamdani et al., 2011). Reported effective concentrations in fruit-crop studies generally fall within the sub-millimolar to low-millimolar range. However, the appropriate dose depends on the PA, application method, crop, tissue, and stress condition. For example, researchers applied 0.25 mM Spd as a foliar treatment to tomato seedlings under saline-alkaline stress, while citrus *in vitro* plants were pretreated with 1 mM Spm before dehydration stress (Li et al., 2015; Shi et al., 2010). In postharvest studies, strawberry fruits were dipped in 0.3–2 mM Put, with 1 and 2 mM treatments producing the strongest extension of storage life, whereas plum fruits were treated with 1 mM Put (Khosroshahi et al., 2007; Serrano et al., 2003). Thus, the effective concentrations reported in these studies range from approximately 0.25 to 2 mM, but this range should not be interpreted as a universal optimum for all fruit crops. However, excessive PA levels can increase catabolism-derived ROS and may become harmful if antioxidant capacity is insufficient. Therefore, field applications need crop-specific dose optimization, stress-specific timing, and integration with other management practices. The specific concentrations, application pathways, target stresses, and physiological outcomes of these exogenous treatments across key fruit crops are summarized in **Table 1**.

**Table 1:** Summary of exogenous polyamine treatments and endogenous polyamine modulation under abiotic stress and during postharvest storage in key fruit crops.

Fruit crop species	Applied PA & concentration	Application method & timing	Target stress	Key physiological, photosynthetic & quality outcomes	References
Tomato (*Solanum lycopersicum*)	Putrescine (Put), 1.0 mM (1000 μM)	Treatment of 30-day-old plants for three consecutive days	Salinity (250 mM NaCl)	Enhanced SOD, CAT, APX, and GR activities; modulated ACC synthase (ACS)/ethylene-related and ion-homeostasis-related gene expression (NHX1, SOS1, HKT1;2); reduced salinity-associated injury.	Rao et al. (2025)
Tomato (*Solanum lycopersicum*)	Spermidine (Spd) 0.25 mM	Foliar spray; applied with 75 mM saline-alkaline stress (Li et al., 2015) or as a 24-h pretreatment before 300 mM saline-alkaline stress (Yang et al., 2022).	Saline-alkaline stress (mixed NaCl/Na_2_SO_4_/NaHCO_3_>/Na_2_CO_3_>; study-dependent concentration)	Protected chloroplast antioxidant systems, chloroplast ultrastructure, and chlorophyll metabolism (Li et al., 2015); induced SlPAO4/PAO-dependent H_2_O_2_ signaling and enhanced SOD, CAT, APX, and GR responses (Yang et al., 2022).	Li et al. (2015); Yang et al. (2022)
Tomato (*Solanum lycopersicum*)	Spermine (Spm), 0.5 mM	Root-zone watering (0.5 L per plant once daily for 7 days) before stress	Combined salinity and paraquat stress (150 mM NaCl + 1.5 μM paraquat)	Reduced leaf damage, H_2_O_2_, and MDA accumulation; enhanced survival, growth, net photosynthetic rate, and PSII function under combined stress.	Pascual et al. (2023)
Citrus (*Citrus reticulata* ‘Red Tangerine’)	Spermine (Spm) 1.0 mM	Pretreatment of *in vitro* plants before dehydration	Dehydration	Reduced wilting, water loss, and electrolyte leakage; increased endogenous PA accumulation and ADC and SPMS expression; enhanced SOD and POD activities; promoted stomatal closure.	Shi et al. (2010)
Grapevine (*Vitis vinifera*)	Endogenous PA modulation via ABA signaling	Drought-induced endogenous metabolic reorientation	Drought	ABA was associated with cultivar-dependent reorientation of PA metabolism; soluble Put and Spd increased strongly in the drought-tolerant genotype, whereas Spm accumulated in the sensitive genotype; ABA also promoted PA exodus to the apoplast, linking PA oxidation with H_2_O_2_-mediated stress signaling.	Toumi et al. (2010)
Apple (*Malus domestica*)	Stress-induced endogenous Put; exogenous Put at 1 and 5 mM	Apple callus exposed to 200 mM NaCl; exogenous Put supplied in the culture medium during salt treatment	Salinity (200 mM NaCl)	Salt stress increased free Put together with MdADC expression and ADC activity, while ODC showed a contrasting pattern; exogenous Put (1 or 5 mM) increased fresh-weight recovery and significantly reduced electrolyte leakage under salinity.	Liu (2006)
Pear (*Pyrus communis*)	Spermidine (Spd) via transgenic overexpression of MdSPDS1	Transgenic European pear shoots overexpressing the apple spermidine synthase gene MdSPDS1	Salinity (150 mM NaCl) and hyperosmotic stress (300 mM mannitol)	Increased endogenous Spd and stress tolerance; under NaCl or mannitol, transgenic shoots showed higher antioxidant enzyme activities and lower H_2_O_2_ and MDA accumulation than wild type.	Wen et al. (2008); He et al. (2008)
Mango (*Mangifera indica*)	Spermine (Spm) 0.01 mM; spermidine (Spd) 0.5–2.0 mM; putrescine (Put) 1.0 mM (study-dependent)	Postharvest fruit treatment before storage	Postharvest ripening and storage deterioration	Delayed softening, color development, weight loss, and deterioration; maintained fruit firmness and selected quality attributes. In ‘Langra’ mango, 2 mM Spd was among the most effective treatments for maintaining firmness and delaying deterioration during storage.	Malik et al. (2006); Zahedi et al. (2019)
Strawberry (*Fragaria × ananassa ‘Benihoppe’*)	Endogenous Put/PA modulation; exogenous Put/ABA and biosynthesis inhibitors used mechanistically	Detached-leaf, PEG 6000, and pot-drought experiments; qRT-PCR, western blotting, RNA-seq, physiological and pharmacological analyses	Drought/water deficit	Early drought promoted FaADC1-associated Put and FaNCED1-associated ABA accumulation; later ABA reprogrammed PA homeostasis (↓FaADC1; ↑FaSAMDC1/FaPAO5). Put-associated responses included photosynthesis and oxidative phosphorylation, with stomatal and hormone crosstalk linking ABA and PAs.	Gao et al. (2024)
Strawberry (*Fragaria × ananassa*)	Putrescine (Put), 0.3, 0.5, 1.0, or 2.0 mM (1.0–2.0 mM most effective)	5-min postharvest immersion before storage at 5 °C	Postharvest storage and senescence	Reduced ethylene production and fruit softening; maintained firmness and marketable quality; extended storage life, with 1.0 and 2.0 mM Put giving the strongest effects.	Khosroshahi et al. (2007)
Plum (*Prunus salicina*)	Putrescine (Put), 0.1–2.0 mM (1.0 mM used in the multi-cultivar study)	Postharvest infiltration/dipping or preharvest spray before storage	Postharvest mechanical injury, ripening, and low-temperature storage	Reduced mechanical damage and ethylene production; delayed softening and ripening; maintained fruit firmness and extended storage life across several plum cultivars.	Pérez-Vicente et al. (2002); Serrano et al. (2003); Khan et al. (2007)

Postharvest applications also have value, especially in strawberry, mango, and plum, where PAs can help maintain antioxidant capacity, firmness, and shelf life (Serrano et al., 2003; Tiburcio et al., 2014; Zahedi et al., 2019). These effects matter for fruit quality, but they should be distinguished from photosynthesis-related mechanisms, since harvested tissues no longer reflect active whole-plant carbon assimilation.

For field use, PA applications should be evaluated using realistic agronomic endpoints. A treatment that improves the F_v_/F_m_ ratio under controlled stress conditions is promising, but growers ultimately need improvements in fruit set, yield stability, fruit size, soluble solids, acidity, color, shelf life, or marketable yield. Therefore, future field trials should connect physiological responses with practical production outcomes (Manzoor et al., 2023). In perennial systems such as citrus, grapevine, apple, and mango, repeated seasonal applications should also be tested because stress responses may vary with phenological stage, canopy age, and accumulated field stress (Grossman, 2023).

Application strategy may also differ among crops. Foliar Spd or Spm may be useful when the main target is leaf-level photoprotection during heat or drought, while root-zone treatments may better influence water status, rhizosphere stress responses, or early systemic signaling (Hai et al., 2022; Pascual et al., 2023). Put treatments may be particularly relevant for stomatal regulation because DAO-derived H_2_O_2_ is involved in guard cell signaling. However, because high PA concentrations can increase ROS through catabolism, dose-response experiments are essential. The practical goal is to identify a treatment window in which PA-derived signaling is sufficient to prime defense but not high enough to intensify oxidative stress.

## 8 Synthesis, research gaps, and future directions

The evidence reviewed here supports an integrated PA–antioxidant–photosynthesis axis. Under abiotic stress, hormonal and stress-responsive pathways activate PA biosynthesis. The resulting PA accumulation strengthens antioxidant defense, both directly and through DAO/PAO-derived H_2_O_2_ redox priming. Improved antioxidant capacity then helps maintain redox balance and protects key photosynthetic processes, including thylakoid membrane stability, PSII efficiency, chlorophyll retention, stomatal regulation, and carbon fixation (Foyer and Shigeoka, 2011; Gururani et al., 2015). This framework is strongly supported by physiological evidence from fruit crops and molecular evidence from *Arabidopsis*. However, the mechanistic connection between these two systems remains incomplete, particularly at the level of gene regulation in fruit crops.

Six major research gaps are especially important for advancing this field. The first gap is the regulation of the Calvin cycle in fruit crops. No study has yet tested whether PA treatment upregulates Calvin cycle-related genes, such as Rubisco, GAPDH, FBA, SBPase, or related targets, in any fruit crop. The second gap is transcription factor identification. The transcription factors that perceive or transmit PA-derived H_2_O_2_ signals and activate antioxidant- and photosynthesis-related genes in fruit crops remain unknown. The third gap is simultaneous multi-endpoint analysis. Very few studies measure PA levels, antioxidant enzyme activities, PSII efficiency, gas exchange, and Calvin cycle activity in the same crop, under the same stress condition, and within the same experimental design.

The fourth gap is the characterization of PA catabolism gene families. PAO gene families are relatively well-resolved in *Arabidopsis* and partially characterized in tomato, but comparable information remains limited for citrus, grape, strawberry, mango, and other fruit crops. The fifth gap is chloroplast localization of PA biosynthesis. ADC localization has been demonstrated in model systems, but direct evidence in fruit crops using organelle fractionation, immunodetection, or fluorescent reporter lines remains lacking. The sixth gap is PA transport and compartmentation. Although PUT2-mediated shoot-to-root PA transport has now been functionally demonstrated in tomato, transporter identity, substrate specificity, organelle transport, and regulatory mechanisms remain poorly characterized across other fruit crops (Yang et al., 2025). Addressing these gaps will be important for moving the field from physiological observation toward a clearer molecular understanding of PA-mediated photosynthetic stress tolerance in fruit crops.

Future research should move from descriptive physiology toward causal molecular testing. Priority experiments include time-resolved RNA-seq in PA-treated, stressed fruit crops; qPCR validation of antioxidant and Calvin cycle genes; enzyme assays for Rubisco and SBPase activities; chlorophyll fluorescence and gas exchange measurements in the same plants used for RNA-seq; and promoter studies to identify PA-responsive transcriptional regulators. CRISPR, virus-induced gene silencing, transient promoter-reporter assays, and PAO/DAO inhibitor studies could help determine whether PA-derived H_2_O_2_ is required for the activation of antioxidant and photosynthetic genes.

Research should also extend beyond single, constant-stress treatments, as fruit crops under field conditions are often exposed to multiple stresses, either simultaneously or sequentially. Consequently, PA responses may vary depending on whether drought, heat, salinity, excess light, oxidative stress, and subsequent recovery occur alone, in combination, or one after another. Future experiments should therefore test whether PA-derived H_2_O_2_–Ca²^+^/MAPK signaling, hormone crosstalk, GABA/carbon–nitrogen metabolism, and PA transport are conserved under combined stresses and across stress/recovery phases. Such designs are particularly important because a signaling-level PA dose that primes defense under one condition may become damaging when PA oxidation and ROS production are superimposed on another stress (Mittler, 2006; Pascual et al., 2023; Yang et al., 2024).

The most informative experimental design would include at least five endpoints measured in the same plants: endogenous PA levels; DAO/PAO activity or gene expression; antioxidant enzyme activities; chlorophyll fluorescence and gas exchange; and Calvin cycle enzyme expression or activity. Such a design would allow researchers to test sequence and causality across the axes. For example, if PA accumulation precedes H_2_O_2_ signaling, antioxidant gene activation, improved Fv/Fm, and maintenance of Rubisco or SBPase activity, the model in **Figure 3** would gain strong mechanistic support. If these steps do not occur in sequence, the model would need to be revised for specific crops or stress conditions.

## 9 Conclusion and future perspectives

Polyamines (PAs) are important regulators of abiotic stress tolerance in fruit crops because they connect metabolic homeostasis, redox signaling, antioxidant defense, and photosynthetic protection. Available evidence shows that stress-induced PA accumulation or exogenous PA application can enhance antioxidant enzyme activity, reduce lipid peroxidation and membrane injury, preserve chlorophyll, protect PSII, and improve stomatal regulation and plant water status. Together, these responses support the proposed PA–antioxidant–photosynthesis axis as a useful framework for explaining how fruit crops maintain physiological function under drought, salinity, heat, cold, and oxidative stress. However, most evidence for fruit crops remains physiological or biochemical, while direct molecular and causal validation remains limited.

Future research should move beyond measuring individual stress indicators and adopt integrated, time-resolved experimental designs. Endogenous free, conjugated, and bound PA fractions should be measured together with the expression and activity of PA biosynthetic and catabolic enzymes, DAO- or PAO-derived H_2_O_2_ production, antioxidant responses, chlorophyll fluorescence, gas exchange, and Calvin-cycle activity in the same plants. Attention should be given to determining whether PA treatments directly regulate genes and enzymes involved in carbon fixation, including Rubisco, Rubisco activase, GAPDH, FBA, SBPase, and FBPase. Identifying the transcription factors, promoter elements, and signaling pathways that link PA-derived redox signals to antioxidant- and photosynthesis-related genes will be essential for establishing causality. Transcriptomics, proteomics, metabolomics, gene silencing, CRISPR-based approaches, promoter assays, enzyme inhibitors, and organelle-localization studies will help clarify these mechanisms.

The translation of PA-based treatments into fruit production will also require greater attention to crop species, genotypes, tissues, developmental stages, stress combinations, treatment timing, application methods, and PA concentrations. This is especially important because controlled PAs oxidation may promote protective signaling, whereas excessive PA catabolism can increase ROS production and aggravate oxidative damage. Long-term field trials in perennial fruit crops should therefore connect molecular and physiological responses with practical outcomes such as flowering, fruit set, yield stability, fruit quality, and marketable production. By integrating mechanistic studies with realistic field evaluation, future research can determine whether PA manipulation represents a reliable strategy for improving the resilience, productivity, and quality of fruit crops under increasingly variable climatic conditions.
